# Antimicrobial Solutions for Endotracheal Tubes in Prevention of Ventilator-Associated Pneumonia

**DOI:** 10.3390/ma16145034

**Published:** 2023-07-17

**Authors:** Lavinia Marcut, Veronica Manescu (Paltanea), Aurora Antoniac, Gheorghe Paltanea, Alina Robu, Aurel George Mohan, Elena Grosu, Iuliana Corneschi, Alin Danut Bodog

**Affiliations:** 1 Faculty of Medicine and Pharmacy, University of Oradea, 10 P-ta 1 December Street, RO-410073 Oradea, Romania; 2 Intensive Care Unit, Clinical Emergency Hospital Oradea, 65 Gheorghe Doja Street, RO-410169 Oradea, Romania; 3 Faculty of Material Science and Engineering, University Politehnica of Bucharest, 313 Splaiul Independentei, District 6, RO-060042 Bucharest, Romania; 4 Faculty of Electrical Engineering, University Politehnica of Bucharest, 313 Splaiul Independentei, District 6, RO-060042 Bucharest, Romania; 5 Department of Neurosurgery, Clinical Emergency Hospital Oradea, 65 Gheorghe Doja Street, RO-410169 Oradea, Romania; 6 Romfire Protect Solutions SRL, 39 Drumul Taberei, RO-061359 Bucharest, Romania

**Keywords:** antimicrobial coating, biofilm, endotracheal tube, ventilator-associated pneumonia

## Abstract

Ventilator-associated pneumonia is one of the most frequently encountered hospital infections and is an essential issue in the healthcare field. It is usually linked to a high mortality rate and prolonged hospitalization time. There is a lack of treatment, so alternative solutions must be continuously sought. The endotracheal tube is an indwelling device that is a significant culprit for ventilator-associated pneumonia because its surface can be colonized by different types of pathogens, which generate a multispecies biofilm. In the paper, we discuss the definition of ventilator-associated pneumonia, the economic burdens, and its outcomes. Then, we present the latest technological solutions for endotracheal tube surfaces, such as active antimicrobial coatings, passive coatings, and combinatorial methods, with examples from the literature. We end our analysis by identifying the gaps existing in the present research and investigating future possibilities that can decrease ventilator-associated pneumonia cases and improve patient comfort during treatment.

## 1. Introduction

### 1.1. General Considerations

An important problem of hospital-acquired infections or nosocomial infections is foreseen because today’s devices made from biomaterials are much more frequently used to stabilize the health state of critically ill or injured patients [1]. One of the most frequent infections is considered to be ventilator-associated pneumonia (VAP). It is related to the mechanical ventilation (MV) process and invasive tracheal intubation procedure [2,3,4,5]. Other infections encountered in hospitals are catheter-associated urinary tract infections, central line-associated bloodstream infections, and surgical site infections [6,7]. The use of indwelling devices generates an unwanted exposure of the human body to the external medium and can be considered a route for exogenous and endogenous microorganism circulation [8,9,10,11].

**Figure 1:**
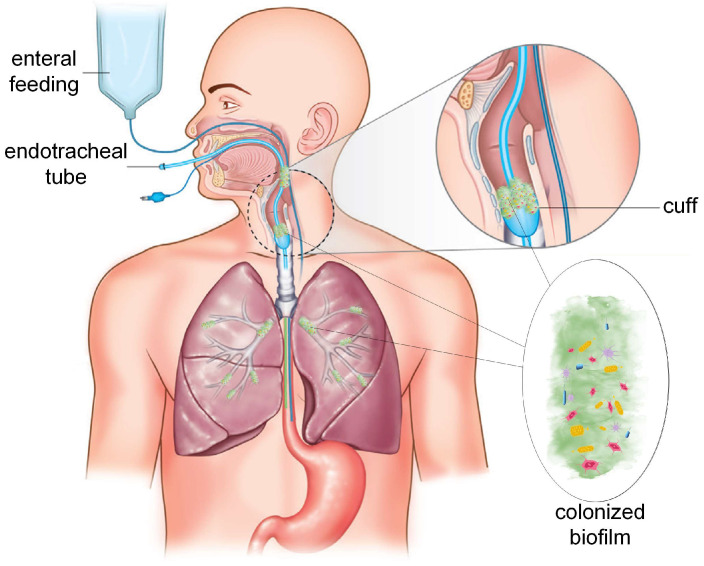
Ventilator-associated pneumonia development based on biofilm formation on the endotracheal tube surface. Adapted from [19] (CC-BY 3.0 in the original reference).

Ventilator-associated pneumonia can be defined as a disease that occurs at least two days after mechanical ventilation is started and affects the pulmonary parenchyma [12,13]. Unfortunately, its first signs are very similar to those observed in other health conditions, so early diagnosis becomes challenging. There are identified in the literature two types of VAP with early and late onset. Alves et al. [14] considered that early-onset VAP occurs at a time comprising between 2 and 4 days post-intubation, and late onset develops at a time interval higher than 5 days post-intubation [15]. In the first case, the duration of hospitalization was estimated at a maximum of 27 days, and for late onset, this parameter was about 36 days [16,17,18,19]. In addition, the incidence rate for early onset was between 11–16%, associated with a mortality rate of about 16–23%, while in the case of late onset, a higher incidence rate of about 84% was identified, and a mortality rate of 44% was reported [20,21]. A study made by the International Nosocomial Infection Control Consortium stated that in the United States of America (USA), the VAP rate was as low as 1 to about 3 episodes per 1000 ventilator days [22], while in the European Union, a rate of 18.3/1000 ventilator days was reported [23]. In developing countries, this quantity was estimated at 22 episodes per 1000 ventilation days [24]. The main risk factors considered were the prolonged time of MV, airway bacterial colonization, micro aspiration, the compromised immune systems of the patients, and impaired mucociliary clearance (Figure 1) [14]. Due to the COVID-19 pandemic situation, the incidence rate of VAP in intensive care units (ICU) increased to 26 cases per 1000 ventilator days from 15.4 cases per 1000 ventilator days before COVID-19. Still, although the worldwide pandemic seems to be ending, patients admitted to ICU with medical treatment needing MV are at a high risk of VAP. There is a direct influence relating to patient age, associated comorbidities such as cancer or other chronic obstructive pulmonary diseases, longer need for MV and ICU care, and overall health, that can lead to mortality or disability [14]. Treatments for all these exhibit high costs. Tawfig et al. [25] and Zimlichman et al. [26] analyzed the healthcare costs associated with VAP and found that they comprised between USD 28 and 33 billion per year in the USA, an amount that represents about 32% of all hospital-acquired infection. In [27,28,29], the economic burden of VAP in European countries was estimated between EUR 13 and 24 billion per year.

**Figure 2:**
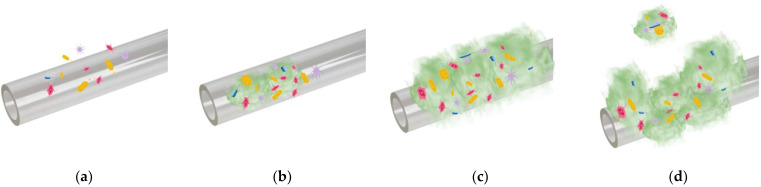
Stages of complex biofilm formation on ETT surface: (**a**) attachment phase, in which bacteria change their state from planktonic to sessile; (**b**) establishment phase characterized by secretion of biofilm extracellular matrix; (**c**) development and maturement of the biofilm; (**d**) biofilm dispersion and disassembly.

The most common pathogens involved in biofilm formation on medical devices associated with VAP are antibiotic-sensitive bacteria such as methicillin-sensitive *S. aureus*, *Haemophiles influenzae*, *Streptococcus pneumoniae*, *Escherichia coli*, *Serratia* spp., *Klebsiella pneumoniae*, *Proteus* app, and multidrug-resistant or antibiotic-resistant bacteria such as methicillin-resistant *S. aureus*, *Pseudomonas aeruginosa*, *Enterobacter* spp., *Acinetobacter* spp., and Vancomycin-resistant *Enterococcus* [14,30,31]. The biofilm can be defined as an aggregate of different bacteria species covered in an extracellular polymeric matrix made of carbonate-rich polymers such as cellulose or alginate and proteins such as nucleic acids or amyloids (Figure 2) [32]. Usually, bacteria are characterized by phenotypic plasticity, which permits them to modify their gene expression that determines a planktonic state, in which they float free and are subject to colonization and motility, or a sessile state with enhanced metabolism and reproduction functions [33]. Bacteria that are in the sessile state can create a biofilm because they have reduced motility. The first step in biofilm formation is pathogen attachment that can be linked through macromolecules on the bacteria’s surface or inside their systems, such as lipopolysaccharides or exopolysaccharides [34]. After some time, the connection between bacteria and the medical device becomes stronger, and biofilm formation begins. The bacterial interaction and number increase while the biofilm matures, and a scaffold-like structure occurs [33,34]. The high density of cells enhances quorum sensing, determines a decrease in cellular division, and reduces metabolic activity [35]. In the case of increased phenotypic variations between bacteria, progression or defense actions can appear directly linked to antibiotic resistance [36]. When the cell density reaches a certain value, some cells can leave the biofilm, changing their state into a sessile one and exhibiting high virulence and antibiotic resistance. In some cases, these cells can again enter a planktonic state and populate and infect other parts of the human body by initiating biofilm formation in other places [37,38]. It has been proved that due to the biofilm complexity and the multitude of bacteria species, 10–5000 times more drugs are needed to destroy the biofilm compared to a free-living bacterial infection [39]. In clinical practice, when indwelling medical devices are necessary to maintain or prolong the patient’s life, attention should be devoted to hindering biofilm formation for as long as possible.

### 1.2. Endotracheal Tube a Life-Saving Medical Device with Negative Impact on Patient Health

**Figure 3:**
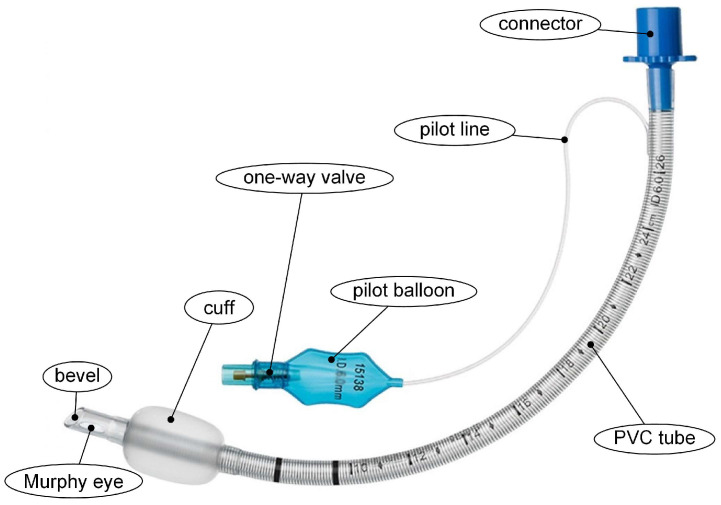
Architecture of a standard ETT.

Mechanical ventilation requires using endotracheal tubes (ETTs) for the patient intubation procedure. Hashemi et al. [40] estimated that annually about 50 million patients undergo intubation with ETTs for a short time during surgery or for a longer period if they have a severe illness and their breathing ability is lost. Since ETTs are considered life-saving medical devices, they are also a platform that is beneficial for biofilm formation. Usually, EETs are made of non-degradable and highly biocompatible polymers such as polyvinyl chloride (PVC) or poly(dimethylsiloxane) (PDMS), but other materials such as silicone, rubber, or metal can be involved [41]. In Figure 3 is presented the standard anatomy of an endotracheal tube. It contains a marking of length expressed in centimeters that helps the clinicians to introduce the tube inside the human body and carefully control its movements. Also, a continuous radiopaque marking is embedded along the tube length to permit X-ray identification in the chest area and to estimate the appropriate depth of the medical device [42,43,44]. Usually, the ETT is introduced on the right side of the laryngoscope. It has a bevel, which determines a high-quality visualization of the zone ahead of the tube and offers easier passage through the vocal cords. Murphy’s eye is a hole positioned in opposition to the bevel to permit the gas passage if the tube tip is obstructed. Almost all the ETT models exhibit a cuff and an inflatable balloon at the end of the tube. It generates a seal against the trachea wall and prevents fluid and secretions from leaking into the lungs and gas from leaking around the tube material. The cuff is linked to a pilot balloon placed outside the patient’s body and acts as a reservoir to reduce minor variations in cuff pressure. Attached to this balloon, a one-way valve can be observed that prevents gas from escaping the cuff. A standard adapter is always used in order to permit the addition of different anesthesia or respiratory equipment [41].

Endotracheal tubes are directly linked to infections of the lungs because they are prone to tracheobronchial colonization by permitting the free passage of bacteria from the stomach, oropharynx, and sinuses [45]. ETTs hinder swallowing or coughing, which clear the mucus and stop the micro-organisms’ movement to the lower respiratory tract [46]. As a direct consequence, the medical devices are contaminated, and biofilm formation in the outer and inner lumens of the tube becomes possible. The most frequently encountered sources of VAP are considered to be aspiration, secretions above the ETT cuff, and intubation procedure due to contamination of equipment. In some cases, as described in Section 1.1., portions of the formed biofilm can dislodge and be introduced directly into the patient’s lungs, leading to VAP [47]. Another complication of ETT use is that during the tube insertion, a certain pressure is exerted onto the trachea and larynx, and foreign body responses such as inflammation or local irritation may be observed. In some cases, unwanted post-extubation obstructions occur [3].

The airway anatomy of pediatric patients is different from the adult respiratory tract. Firstly, the head of a child is larger in comparison with body size, exhibiting a prominent occiput, a fact that can determine airway obstruction because the neck is flexed when the young patient lies on a flat surface [48,49]. Secondly, the mandible is shorter, the tongue is larger, and prominent tonsils and adenoids are present in the case of preschoolers [50]. All the factors mentioned above have an important influence on reducing the upper airway space, which can make mask ventilation or laryngoscopy difficult. If hypnotic or anaesthetic drugs are used, a loss of upper airway muscle tone can appear, and the risk of upper airway obstruction increases. Another anatomical difference is that the hypopharynx has a narrow width and reduced height since the larynx is higher in the neck, and sometimes the mandible is positioned in line with the upper glottic structures [48]. Vocal cords are not placed at 90° to the trachea, and the intubation procedure must be conducted carefully. The children’s epiglottis is U-shaped, and the trachea’s flexible cartilaginous rings can obstruct negative pressure ventilation [51]. The CDC and Healthcare Infection Control Practices Advisory Committee recommend an ETT with a dorsal lumen to permit the easy drainage of orotracheal and respiratory secretions [52]. However, uncuffed ETTs usually used in neonates generate an increased risk of VAP since cuffed ETTs are linked to a decreased necessity of ETT changes and post-extubation stridor but increase the number of days with mechanical ventilation [53,54,55].

VAP is a serious life-threatening condition which can lead to sepsis [56,57], organ damage, and endotoxin-driven inflammation [58]. In the case of old or newborn patients, delirium or brain inflammation can occur. In order to prevent VAP occurring, some management guidelines have been established. These include reduced duration of MV procedure concomitantly with a decreased dose of sedation drugs, treatment of pressure ulcers, prophylaxis of venous thrombosis, and a semi-recumbent body position [12]. If these procedures are applied during the first five days of MV, they show high efficiency only in the case of early-onset VAP [59,60]. As we have underlined before, VAP cases are polymicrobial infections, and they require dedicated treatment. Unfortunately, in most cases, due to the plethora of species involved in biofilm formation, the treatment is nonspecific and inefficient, being directly linked to a high mortality rate.

Many researchers consider VAP prevention much more important than its treatment, and adequate approaches are continuously sought. One proposed solution consists of ETT surface modification to promote antibacterial properties. In some cases, active materials with antibacterial properties were applied onto the ETT surface, which permitted a direct link between them and the dangerous bacteria. Other authors investigated passive surface modifications by changing the topography of the surface to a nanostructured and superhydrophobic one, hindering in this way bacterial adhesion. Other researchers considered metal coatings, surfactants, antimicrobial peptides, or photodynamic therapies important in VAP prevention.

In this review paper, we summarize some of the most frequently used techniques for adapting the surface of ETTs to inhibit bacterial colonization and biofilm formation and reduce the number of patients with VAP in ICUs. There is a lack of products with antibacterial properties on the market that have received FDA approval. We found only one manufacturer Bard Medical Division (Covington, GA, USA) that produces Agento^®^ I.C. ETT made from PVC and uses silver ions to interrupt the cellular functions of the bacteria to prevent biofilm formation [61,62], and three other producers (Sharklet Technologies Sharklet^®^ ETT—main material PVC with the textured surface [63]; Bactiguard Holding AB Bactiguard^®^ Infection protection (BIP) ETT—main material PVC with noble metal coating Ag-Pd-Au [64]; N8 Medical Cerashield^TM^ ETT—main material PVC with synthetic antimicrobial peptide Ceragenin [40]) that are seeking FDA approval. We consider it very important to develop new solutions for ETT manufacturing, considering the life-threatening conditions related to VAP.

## 2. Modern Technological Advances in Antimicrobial Coatings for ETTs

As previously mentioned, the literature includes descriptions of active technologies that kill the bacteria and microbes on the medical device surface, passive solutions that modify the implant surface and composition to obtain a surface inadequate for micro-organism adhesion, and combinatorial approaches that endow the ETTs with active antimicrobial or passive antifouling properties. Antimicrobial coatings are considered an innovative way to solve the problem of biofilm formation on the ETT surface. In the following sections, we review the most frequently used technologies in clinical and pre-clinical practice.

### 2.1. Active Antimicrobial Coatings

Active antimicrobial coatings include the use of antiseptics or antibiotics incorporated in coatings, ionic or covalently bonded with a polymeric matrix [65,66]. Other approaches involve coatings of noble metal inserted in or coated on polymeric surfaces [67]. Some metallic materials or their oxides, such as silver (Ag), selenium (Se), silver oxide (Ag_2_O), titanium dioxide (TiO_2_), iron oxides (Fe_2_O_3_, Fe_3_O_4_), zinc oxide (ZnO), and copper oxide (CuO), have bactericidal properties and can be used as nanoparticles or ions if the bulk metal is considered unsafe regarding its increased toxicity for in vivo applications [3,68,69,70,71,72]. Lately, due to advances in the nanotechnology domain, there has been a growing interest in ZnO and Ag_2_O coatings due to their excellent antimicrobial activity [67,73,74]. Attention has been devoted to ZnO nanoparticles because of their compatibility with the human body and increased bactericidal effects on Gram-negative and Gram-positive bacteria. Azam et al. [75] compared the antimicrobial efficiency of CuO, Fe_2_O_3_, and ZnO against *E. coli*, *P. aeruginosa, S. aureus*, and *B. subtilis*. They concluded that ZnO nanoparticles exhibited the most pronounced antibacterial effect and Fe_2_O_3_ nanoparticles had the lowest antibacterial activity. In [76], it was shown that Ag nanoparticles presented important antibacterial activity even though they were used in very low concentrations, while ZnO nanoparticles’ efficiency depended on surface area and concentration. If ZnO nanoparticles are used in higher concentrations and larger surface, they provide increased antibacterial efficiency in comparison with Ag nanoparticles [67]. Some toxic effects characterize TiO_2_ nanoparticles, and these are usually used in combination with biopolymers. They exhibit a moderate antibacterial impact, but in some cases, they have proved to be efficient due to formation of reactive oxidative species, which have the potential to damage the specific DNA of the bacteria [77,78].

#### 2.1.1. Antimicrobial Metal Coatings

One of the most used frequently metal coatings that exhibit antibacterial properties is silver. Silver has been investigated for at least 30 years and was successfully applied to urinary catheters. Now it is being investigated as a coating for ETTs, and because it has succeeded in numerous clinical trials, it has now been commercialized [62,79]. Its action mechanism is based on ionic bindings formed between metal and bacteria cells by increasing the cell permeability, facilitating cell membrane penetration, changing protein activity, and inducing oxidative damage [3]. Many studies reported in the literature have analyzed the antibacterial effects of silver alloys’, silver-based compounds, or nanoparticles when applied as coatings for ETTs. Bechtold et al. [80] synthesized silver nanoparticles (AgNPs) to be used as a biocidal agent in polyurethane coating. They tested the antimicrobial efficiency and activity of the coating according to JIS Z 2801:2000 [81] against *E. coli* (ATCC 8739) and *S. aureus* (ATCC 6538). An antimicrobial activity equal to 2.72 and 3.03 in terms of average numbers of viable cells of *S. aureus* (40 CFU/mL) and *E. coli* (270 CFU/mL) was reported for the silver nanoparticle system. It was concluded that a positive impact was achieved regarding resistance and protection against bacteria. Cruz-Pacheco et al. [82] prepared a coating of polyetheretherketone film with silver nanoparticles and tested its antibacterial activity against *E. coli*, *Serratia marcescens*, and *Bacillus licheniformis*. The antibacterial activity of silver nanoparticles deposited in PEEK films varied as a function of silver nanoparticle concentration and the number of layers. Maximum values were achieved for PEEK with a 0.12 mol/L Ag NP concentration applied in two layers as follows: 2.7 ± 0.3 (*E. coli*), 1.2 ± 0.3 (*S. marcescens*), and 1 ± 0.2 (*B. licheniformis*). It was observed that the concentration of silver is very important for the bacteria’s cellular replication. Derakhshi et al. [83] developed an innovative antibacterial platform based on shape-selective silver nanostructures decorated with amine-functionalized graphene. They noticed that the triangular shape of AgNPs exhibited the highest antibacterial activity against *E. coli* and *S. aureus* with inhibition of 100% for *E*. *coli* and 70% in the case of *S. aureus* at a triangle concentration of 1000 μg/mL. Zhang et al. [84] made an antibacterial coating based on waterborne polyurethane containing silver nanoparticles dispersed in it. An antibacterial rate of 99.99% against *E. coli* and 87.5% against *S. aureus* was found. It was concluded that addition of silver determined a very high antibacterial rate and exhibited important antibacterial properties.

**Figure 4:**
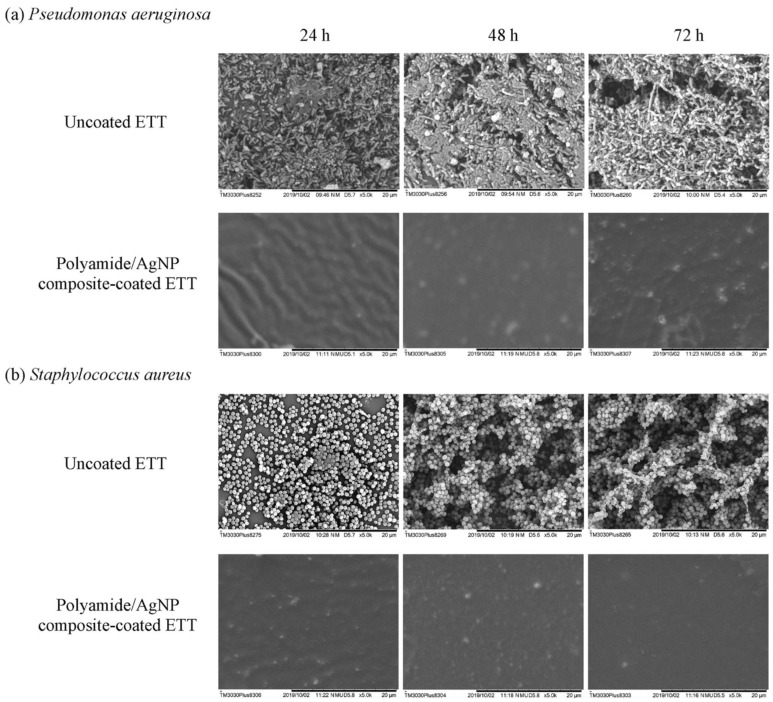
SEM images of biofilm formation: (**a**) *P. aeruginosa* and (**b**) *S. aureus* in the case of uncoated and polyamide/AgNP composite-coated ETTs at times between 24 h and 72 h (magnification 5000×) [85]. Reprinted from [85]. Copyright (2023), with permission from Taylor & Francis.

**Figure 5:**
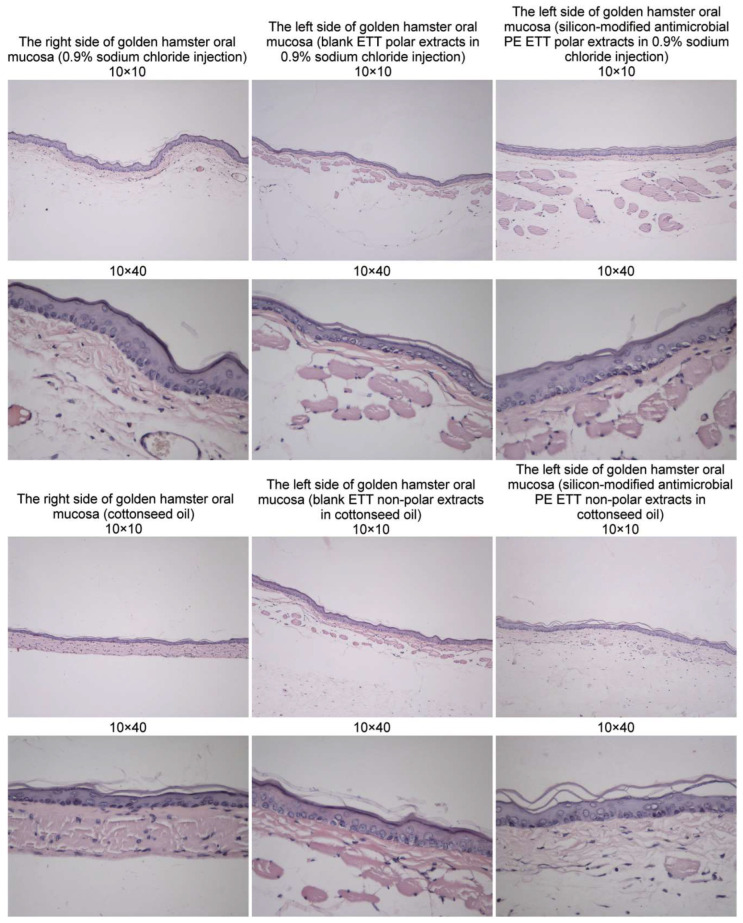
Oral mucous irritation tests in the golden hamster animal model for the bare and Ag-SiO_2_-modified polyethylene ETTs [88]. Figure is licensed under CC-BY 4.0.

Lethongkam et al. [85] investigated a novel polyamide/silver nanoparticle composite coating for a commercially available ETT by testing its inhibitory effects against different species of bacteria, including *C. albicans*, *P. aeruginosa*, *K. pneumoniae*, MRSA, *S. aureus* with the help of an in vitro growth model. They concluded that the coated ETTs exhibited high power in reducing microbial adhesion and planktonic growth in mixed and single-species cultures. Additionally, the inhibition of biofilm formation was noticed after 72 h in the case of *S. aureus* and *P. aeruginosa*, with broad-spectrum activity against Gram-negative and Gram-positive bacteria. The time–kill study showed a decrease of over 99% in viable cells of *P. aeruginosa* within 2 h of incubation. In the case of *S. aureus*, the bacteriostatic effect of the coated ETT reduced by 99.9% the number of viable bacteria cells after 24 h. Figure 4 presents the fact that pathogens highly colonized the uncoated ETTs after one day of incubation. Large amounts of *P. aeruginosa* colonization were observed (Figure 4a), and grapelike colonies of *S. aureus* indicated the biofilm formation process (Figure 4b). Olson et al. [86] studied the antibacterial influence of silver ions on a commercial ETT using a dog animal model. The in vivo study proved that the modified ETT was efficient against *P. aeruginosa*. Also, reduced lung inflammation was reported due to a delay of about a day and a half in bacterial colonization of the ETT. The authors concluded that the coating made an important contribution to the decrease in attached bacterial numbers. Loo et al. [87] analyzed an innovative coating for ETTs comprised of silver nanoparticle–polyvinyl alcohol hydrogels with anti-biofilm activity. They investigated its efficiency on *P. aeruginosa* and *S. aureus* using in vitro models and observed a decrease in biofilm formation for the two bacteria species, and no toxicity was reported against lung cells. Jiang et al. [88] used a silver–silicon dioxide coating on a polyethylene ETT and studied its antibacterial properties based on two in vivo models (golden hamster and rabbit). They observed that in the first animal model, no oral mucosa irritation occurred (Figure 5) since, for the rabbit animal model, no pyrogenic effects were detected. It was concluded that the coating was highly biocompatible with the red blood cells.

In 2015, a noble metal alloy (NMA) made from silver–gold–palladium was developed by Bactiguard^®^ Infection Protection (BIP) to be used as an antibacterial coating for ETTs. Björling et al. [64] performed a randomized clinical evaluation study on this type of coating applied on ETTs, involving 20 patients, in whom BIP ETTs were used. The results were compared to those obtained for 10 patients that were intubated with standard ETTs. Minor differences between the control and BIP ETT groups were noticed. Two patients exhibited dry mouth and cough, but no mucosal damage was reported after a bronchoscopy was performed for all the patients in the clinical trial. Short-term intubation of 5 h was chosen, and a low level of bacterial colonization with normal flora was put in evidence in both cases. The main conclusion of the study was that the BIP ETT was very well tolerated by the patients during a short intubation time. Damas et al. [89] conducted a multi-center, randomized, double-blind study on 323 patients, of whom 168 were included in the NMA-coated group and 155 in the control group. It was observed that VAP occurred in the cases of 18 patients from the control group. The Cox proportional hazards regression method was applied and proved that a delay in VAP occurrence was present in the case of the NMA-coated group. Also, the clinical trial evidenced that the number of antibiotic days was decreased for the NMA-coated group with a value of about 59%. By analyzing tracheal colonization, bacterial occurrence was observed in the cases of 34% of patients from the control group and 30% in the NMA-coated group. The clinical trial concluded that NMA-coated ETTs are a good candidate for FDA approval. Tincu et al. [90] made a randomized controlled trial on 180 patients in a coma state induced by drug abuse who needed MV for a period longer than 48 h. The number of patients was divided into a control group receiving standard ETTs and another group using NMA-ETTs. They found that VAP incidence was 43.16% for the control group and 27.83% for the NMA-coated group. They concluded that in the case of NMA-coated ETT, VAP incidence was reduced concomitantly with days of ventilation.

Other antimicrobial metal coatings include zinc-based [91,92,93], selenium-based [94], and titanium-based [95,96]. Their main attributes and recent literature studies are presented in Table 1.

#### 2.1.2. Antimicrobial Coatings Based on Biocide Impregnation

One of the oldest techniques in antimicrobial approaches consists of incorporating biocidal substances into the polymer before the medical device manufacture [98]. Some interesting studies are presented in the literature. Researchers investigated the in vitro behavior of biocidal application in the case of ETTs. The most frequently used substances include gendine, which can be prepared as a combination between chlorhexidine and gentian violet [99], used alone or combined with gardine. The latter mentioned biocidal is obtained from chlorhexidine and an antiseptic dye [100]. Other studies reported the use of chlorhexidine [101], hexetidine [102], essential oils (i.e., clove oil, eugenol) [103], and coatings that mimic the human body’s defense mechanisms such as nitric oxide (NO)-releasing PVC coatings [104].

**Figure 6:**
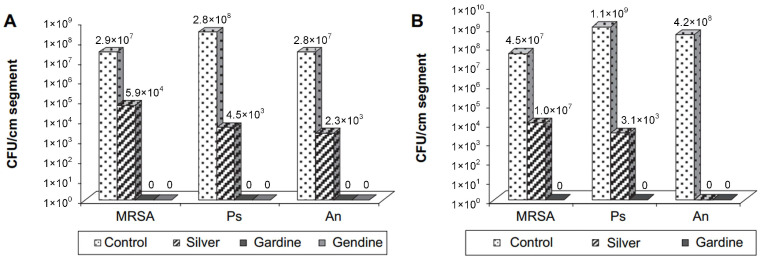
Antimicrobial efficiency of silver-, gardine-, and gendine-coated ETTs. The in vitro adherence of MRSA (Methicillin-resistant *S. aureus*), PS (*P. aeruginosa)*, and An (*A. baumannii*) was studied. After one day, the biofilm cells were dispersed by sonication in neutralizing and non-neutralizing solutions: (**A**) neutralizing solutions: *p* = 0.005 uncoated ETT vs. silver-coated ETT, *p* = 0.003 uncoated ETT vs. gardine-coated ETT (for An *p* = 0.01), *p* = 0.003 uncoated ETT vs. gendine-coated ETT, *p* < 0.003 for silver-coated ETT vs. gendine-coated ETT (for An *p* = 0.01); (**B**) non-neutralizing solutions: *p* < 0.01 uncoated ETT vs. silver-coated ETT, *p* = 0.003 uncoated ETT vs. gardine-coated ETT (for MRSA *p* = 0.004), *p* = 0.003 silver-coated ETT vs. gardine-coated ETT (for An *p* = 0.18) [100]. Reprinted from [100] Copyright (2023), with permission from Elsevier.

Raad et al. [100] investigated the effects of two antiseptic substances, gendine and gardine, used as coatings for ETTs. The authors compared their findings with those obtained from silver-coated ETTs and analyzed the prevention of biofilm colonization. They performed in vitro studies on *S. aureus* (MRSA), *P. aeruginosa*, *A. baumanii*, *C. albicans*, *E. cloacae*, and *K. pneumoniae*. Based on scanning electron microscopy investigations, biofilm formation was not reported in the cases of gardine and gendine-coated ETTs compared with the silver-coated tubes that presented bacteria on their surface. It was concluded that the biocide coatings completely inhibited MRSA, Gram-negative bacteria, and *C. albicans* adherence, and they proved more efficient than silver-coated samples. Furthermore, the antimicrobial activity against the MRSA pathogen was prolonged for 2 weeks (Figure 6).

Chaiban et al. [99] developed a rapid ETT impregnation method based on instant dipping of the device into gendine (GND). The antibacterial activity was investigated in vitro, and strains such as MRSA, *P. aeruginosa*, *E. coli*, and *C. parapsilosis* were included. The cuff of the ETTs was completely coated with GND. It was shown that GND-ETT samples provided high antibacterial properties against MRSA, *P. aeruginosa*, and *E. coli*, exhibiting a prolonged antimicrobial effect extended to 3 weeks. This period is important because an ETT remains inserted into the patient’s body for no longer than 3 weeks. It was concluded that the GND coating was highly efficient in VAP prevention. Jones et al. [102] performed a physiochemical characterization of hexetidine-impregnated ETT and tested its efficiency against *S. aureus* and *P. aeruginosa*. They increased the concentration of hexetidine between 1% and 10% (*w/w*) and observed a decrease in the surface hydrophobicity correlated with an increased micro rugosity. All the samples exhibited good antibacterial properties correlated with increased resistance to microbial adherence. It was noticed that the 1% (*w/w*) hexetidine sample was characterized by the best balance between antibacterial resistance and physiochemical properties and offered a viable solution for reducing the numbers of patients suffering VAP. Venkateswaran et al. [103] used a nanocapsule that slowly released a naturally antimicrobial substance entrapped in a network, representing an innovative coating for ETTs. The nanocapsule was manufactured from poly(lauryl acrylate) and contained eugenol (4-allyl-2-methoxyphenol). The capsule inhibited the growth of *K. pneumoniae* and MRSA and was entrapped in a polymeric coating that released the active substance. It was proved that essential oils are an effective way to fight against biofilm formation.

Homeyer et al. [104] incorporated the nitric oxide (NO) donor S-nitroso-N-acetylpenicillamine (SNAP) into a PVC ETT. Controlled NO release from the SNAP was ensured for 7 days without altering the mechanical properties of ETT. It is well known that SNAP is sensitive to heat exposure. The coating efficacity was tested against *P. aeruginosa*. For the study, it was considered that 23 °C is an adequate temperature for device storage. A reduction in *P. aeruginosa* by 93% in comparison with the control sample was noticed at 24 h. The authors concluded that future long-term bacteria studies and in vivo investigations are necessary to validate the antibacterial effect of nitric oxide, although they performed in vitro investigations, which proved its efficiency.

#### 2.1.3. Bio-Inspired Antimicrobial Coatings

Antimicrobial peptides (AMPs) have been used to generate a surface similar to human tissue and reduce biofilm formation on ETTs. AMPs are polyamino acids with broad-spectrum antimicrobial activity based on amphipathic structures and positively charged elements that bind to the cell membranes, which are negatively charged [105,106]. Recently, AMP coatings with asiolossin-III were applied on ETTs and shown to inhibit bacterial adhesion to the tube surface, exhibiting a low cytotoxic effect on cell lines [107]. Also, new types of compounds have been developed, the so-called ceragenins comprising a cholic acid group coupled with amine groups. These chemical substances do not contain peptides and are characterized by a longer half-life inside the human body. Other advantages exhibited by ceragenins are their low-cost preparation method [108] and their important antimicrobial activity against a large range of pathogens such as MRSA [109], *P. aeruginosa* [110], and *C. albicans* resistant to fluconazole [111].

A novel approach consists of bacteriophage coatings that exhibit the advantages of decreased risk to the human microbiota, self-replication in the body cells, and an efficient method of manufacture [112]. Other investigated coatings are based on biosurfactants such as lecithin, cholesterol, and sphingophasine [113]. These substances have increased emulsifying activity [114] and have proved to be very efficient against bacteria.

In Table 2 are summarized some studies found in the literature regarding bio-inspired antimicrobial coatings [115,116].

### 2.2. Passive Coatings

The surface of the medical device plays an important role in managing microbial adhesion. Properties such as roughness, topography, elemental composition, wettability, and surface free energy could be properly modified to inhibit pathogen attachment [117]. It was reported in the literature that surfaces characterized by moderate wettability are more prone to permit bacterial attachment, while highly hydrophilic or hydrophobic surfaces inhibit this phenomenon [118,119]. Increased surface energy (SFE) is beneficial to bacterial adhesion. Harnett et al. [120] showed that the polar component of the SFE promoted cell development and proliferation if its value was higher than 15 mNm^−1^. On the other hand, the authors demonstrated that in the case of a reduced value for the SFE polar component of about 5 mNm^−1^ or lower, cell spreading was reduced. It is well known that a large surface with significant roughness is favorable to microbial attachment, since one with smooth topography exhibits biofouling properties. Yuan et al. [117] demonstrated that a superhydrophobic surface with a contact angle (CA) higher than 150° was highly resistant to bacterial attachment. They prepared samples with increased roughness and low SFE, where air became entrapped between rough features when a liquid drop came into contact with the surface. These air zones reduced the adhesion force and the contact area between the material and biofilm.

Only a few passive approaches were found in the literature that included nanomodified, hydrophilic/hydrophobic, and micropatterned surfaces.

#### 2.2.1. Nanomodified Surfaces

**Figure 7:**
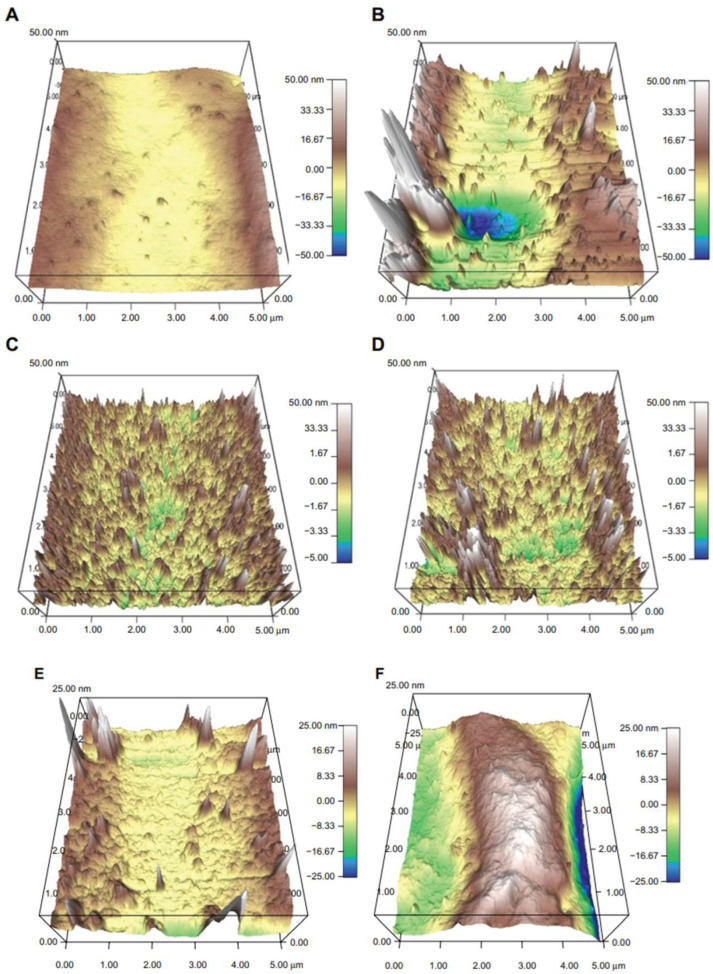
AFM images of topography for control (**A**) and nano-rough surface PVC (**B**), control (**C**,**E**) and nano-rough surface PVC (**D**,**F**) soaked in 10 mM and 100 mM [121]. Figure is licensed under CC-BY NC 3.0.

Today, nanotechnology’s involvement in treating bacterial infections is an important topic of research. Conventional biomaterials do not exhibit nanoscale roughness. In order to influence the bacteria’s behavior, it is necessary to have surface roughness at the nanometer level to interfere with small parts. Durmus et al. [121] combined the antibacterial effect of ETT surface nano-roughness with sugar metabolites such as fructose and noticed a decrease in planktonic *S. aureus* bacteria number by analyzing it in solution or in the biofilm which was formed on an ETT. They concluded that this engineered surface combined with fructose in the absence of an antibiotic substance could be successfully used to reduce biofilm formation and additionally to prevent the growth of antibiotic-resistant bacteria. In Figure 7 are presented atomic force microscopy (AFM) micrographs that put in evidence distinct topographies for control surfaces, in comparison with nano-rough surfaces created using the *Rhizopus arrhizus* lipase.

Machado et al. [122] modified the surfaces of ETTs by soaking the medical devices in a fungal lipase (*Rhizopus arrhisus*). To test the efficiency of the nanometer surface, they used a dynamic airway-condition medium and investigated the concentration and location of bacterial growth on the ETT for 24 h. Their experiments revealed a 1.5 log reduction in the number of *S. aureus*, and the paper’s main conclusion was that the nanomodified surface exhibited increased antibacterial activity in comparison with the conventional ETT because the lipase etching suppressed the pathogen growth by generating a nano-rough surface that proved to be a cheap solution for clinicians to fight against VAP. In other studies [123,124] the same solution of using ETTs etched with *Rhizopus arrhizus* was involved in testing the beneficial effect of the nanostructured surface on *P. aeruginosa*, using an in vitro model of the pediatric airway ventilated for 24 h or classical cytotoxicity tests. A reduction of 2.7 log [123] and about 40% [124] on the modified ETT surface was reported. The authors concluded again that this type of surface modification is efficient against one of the most dangerous pathogens found in hospitals.

Other types of solutions such as controlling the mechanical roughness and the use of polishing techniques resulting in a random texturized surface roughness have not been extensively investigated and much more research must be conducted in this direction.

#### 2.2.2. Hydrophilic/Hydrophobic Surface Characteristics

**Figure 8:**
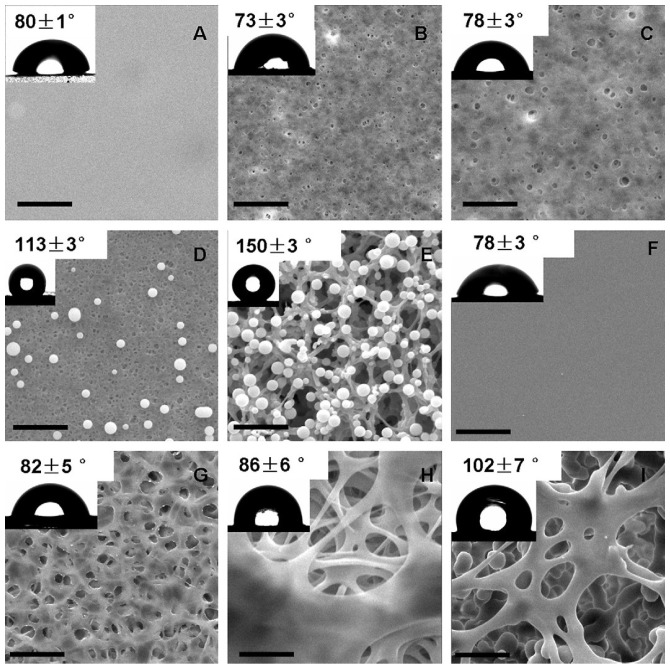
SEM images of PVC samples: (**A**) unmodified PVC; ethanol/methanol-modified PVC (**B**,**F**) 15% (*v*/*v*); (**C**,**G**) 20% (*v*/*v*); (**D**,**H**) 25% (*v*/*v*); (**E**,**I**) 35% (*v*/*v*). Inserts indicate the CA for each surface, and the bar represents 10 μm [126]. Reprinted from [126]. Copyright (2023), with permission from Elsevier.

The relationships established between material surface and pathogens are governed by the surface charge/hydrophobic attributes that are important in the development of biofouling of medical devices. Triandafillu et al. [125] investigated the adhesion of *P. aeruginosa* strains to oxygen-plasma-treated (O_2_ plasma-treated) PVC endotracheal tubes compared with commercial devices. After the O_2_ plasma treatment, the surface became hydrophilic and a reduced adherent bacteria number of 70% was observed. In the case of liquid water, the CA for the curved PVC was about 86.7°; for flattened PVC, it was equal to 85.5°, and in the case of O_2_ plasma-treated PVC, it was found to have a value of 10.2°. Regarding surface roughness, the maximum value was obtained in the case of curved PVC (129 nm), followed by O_2_ plasma-treated PVC (125 nm) and flattened PVC (72.8 nm). The authors concluded that the surface modifications that included hydrophilization of the PVC based on oxygen-plasma treatment are an efficient way to decrease the initial adhesion of bacteria. Unfortunately, this treatment did not provide sufficient power to inhibit biofilm formation completely. For future research, the authors proposed a combined strategy between surface modification and bactericidal or anti-microbial agents. Loo et al. [126] modified the PVC surfaces of medical devices based on a combination of non-solvents, such as ethanol and methanol, and a solvent (tetrahydrofuran). Initially, the surfaces of the commercial devices had a CA of about 80°. The methanol-treated samples exhibited a more hydrophilic character (CA between 78–102° when the methanol concentration varied from 15% to 35% (*v*/*v*)) in comparison with the ethanol-treated PVC (CA between 73–150° as a function of ethanol concentration increase from 15% to 35% (*v*/*v*)) (Figure 8). Two important surface modifications were noticed in the case of the non-solvent treatments: the material surface became porous with different pore shapes and sizes and presented a much more hydrophobic character compared with the untreated samples. The antibacterial effect of the surface modifications was tested against *P. aeruginosa*, and microcolonies were present at 24 h of incubation. The authors concluded that this surface treatment induced a delay in the bacteria colonization process from 18 h (untreated samples) to 24 h (surface-treated samples).

The studies mentioned above demonstrate that surfaces with increased hydrophobicity are beneficial in delaying biofilm formation for a maximum of 24 h, being useful for patients that do not need an increased duration of artificial ventilation.

#### 2.2.3. Micropatterned Surface Modifications

Micropatterning technology has been used in manufacturing well-defined and reproducible microstructures with unique geometry [127]. In this direction, an innovative ETT surface called Sharklet^®^ was developed based on photolithography. This design mimics the placoid scales that characterize shark skin.

Mann et al. [63] investigated the effect of micropatterned endotracheal tubes to reduce secretion-related lumen occlusion. The authors developed in vitro and in vivo models that simulated all the clinical manifestations present in patients with ETT occlusion. The Sharklet^®^ micropatterned ETT was investigated regarding its ability to reduce the accumulation of airway mucus and bacterial biofilm in comparison with commercially available PVC ETTs (airway patency—ETTs made by Medline Industries, Inc., Mundelein, IL and preclinical testing—ETTs from Mallinckrodt^TM^, Medtronic, Mineapolis, MN) and Agento^®^ I.C. silver-coated PVC ETTs. The Sharklet^®^ micropatterned tubes were made through injection molding from Pellethane 2363-90AE thermoplastic polyurethane (TPU, Lubrizol). The in vitro biofilm model was based on a drip flow reactor that generated biofilm formation with *P. aeruginosa*. It was observed that the Sharklet^®^ ETT reduced the biofilm formation by 71% in comparison with the silver-coated ETT, which had 65% reduction. The other study consisted of model analysis of airway patency; after 48 h, the micropatterned surface ETT exhibited a mucus weight decrease of about 86%, 72%, and 69% in the distal, middle, and proximal sections. It was noticed that for mucus localized in the lumen, additional steps such as suctioning were necessary. These maneuvers are dangerous because biofilm detachment becomes possible. The in vivo airway patency model involved animal models such as five female Dorset sheep (24 kg) (two in the control group and three in the treatment group). After one day of intubation, the mucus accumulation was reduced by 61% volume for Sharklet^®^ ETTs in comparison with commercially available ETTs.

**Figure 9:**
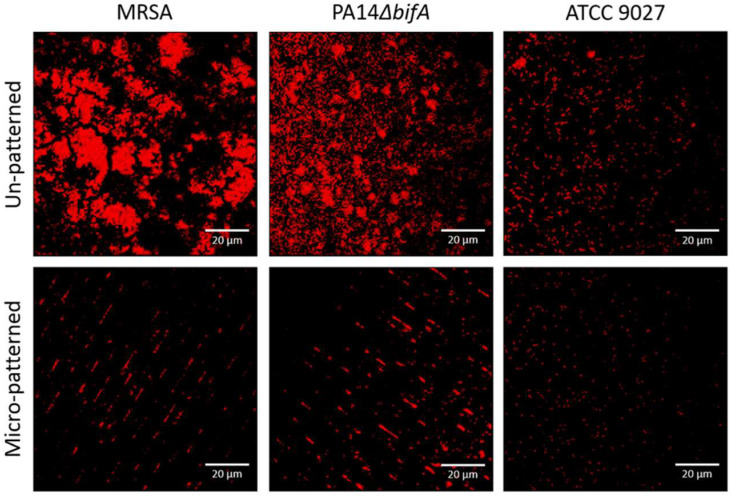
The micropatterned surface efficiency was tested against MRSA and *P. aeruginosa* in comparison with unpatterned ETT. MRSA and PA14Δ*bif*A strains were characterized by 67% (*p* = 0.123) and 52% (*p* = 0.05) median reduction in biofilm compared with the control sample. In the case of *P. aeruginosa* (ATCC 9027) strain, robust biofilm formation was not observed [128]. Figure is licensed under CC-BY 2.0.

May et al. [128] evaluated the Sharklet^®^ surface effect regarding its antiadhesive characteristics against *P. aeruginosa* (multiple strains ATCC10197, ATCC9027, PA14Δ*bif*A), *K. pneumoniae*, MRSA, *A. baumannii,* and *E. coli*. The laboratory-developed strain of *P. aeruginosa* (PA14Δ*bif*A) with Δ*bif*A mutation produced exopolysaccharide in a high amount and rapidly determined the medical device’s colonization and biofilm formation [129]. Micro-patterned and un-patterned control samples were immersed in inoculum with about 107 CFU/mL at room temperature for an incubation time of 1 to 4 h as a function of used the bacteria species’ ability to form a colony. The cell density on the control samples was kept constant, with an average value between 2.5 to 6 logs. It was noticed that in all the cases, the bacterial adhesion was reduced by 96–99.9% for the Sharklet^®^ surface. Supplementary, these samples were immersed for 4 days in a medium that contained the laboratory-modified strain of *P. aeruginosa* and MRSA that facilitated biofilm growth. Reductions of 67% in MRSA and 52% in *P. aeruginosa* biofilm volume were noticed for the micropatterned surface (Figure 9).

Both presented studies put in evidence the efficiency of Sharklet^®^ micropatterning against different types of pathogens, even in the case of those that are very proliferative in biofilm formation. There are just a few studies in the literature that investigate the micropatterned surface effects, and much more research, including in vivo studies, must be carried out to gain a clear image of the antibacterial properties of this innovative design.

### 2.3. Combinatorial Materials

The most important combinatorial materials have active/antibacterial and passive/antibiofouling properties. They are appropriate for the design of new ETT materials because they permit the reduction of drug dose, prevent bacterial attachment and the emergence of drug resistance, and, finally but not least, are characterized by high biocompatibility [130,131]. In Table 3 are presented a selection of the most representative studies found in the literature regarding the active and passive combinatorial strategies.

It can be concluded that combinatorial active/antibacterial and passive/anti-biofouling materials are an effective way to fight against different pathogens responsible for VAP. If these strategies are adopted soon, the burden of the health system regarding intubated patients will be much reduced.

### 2.4. Potential Side Effects and Disadvantages of Antibacterial Coatings

It is well known that simple antimicrobial coatings such as enzymes, phages, antimicrobial peptides (AMPs), nitric oxide (NO), liposomes, and photoactivators can be characterized by a delamination process on the ETT surface [67]. So, as a direct consequence, new active antimicrobial coatings were developed, but unfortunately these solutions exhibit side effects. An innovative approach described in previous chapters consists of AgNPs’ inclusion into ETT coatings. However, the cytotoxicity related to AgNP deposition in the vital organs of patients is a serious concern. The main mechanisms that are linked to the cytotoxicity of silver-based nanoparticles are metal ion release that dissolves the cells and causes metal overload, which activates reactive oxygen species (ROS) [138,139]. Usually, the toxicity of AgNPs is related to their size and preparation method. Today, preferred methods are bio-mediated synthesis [140,141] or other solutions such as the coating of ETTs with a thin layer of noble metal alloy (NMA), which releases a small number of ions and provides so-called Bactiguard infection protection (BIP). Other studies have considered the antibacterial effect of silver nitrate (AgNO_3_) and its related side effects. Maki et al. [142] observed that AgNO_3_ was linked to chemical burns or skin irritations in patients that were treated with silver-nitrate-coated catheters. When using silver nitrate in combination with ETTs, it is important to establish the silver nitrate administration time and concentration to assess its potential toxicity. Pacheco-Fowler et al. [101] recommend an efficient concentration of silver nitrate solution between 0.1% and 1% applied to the inner surface of the ETT for 30 min to 2 h. The potential toxicity of AgNO_3_ is generally related to skin and respiratory tract damage [143]. Al-Sayed et al. [143] used an AgNO_3_ solution with different concentrations between 0.019% and 0.185% with different pH. It was found that samples with concentrations of 0.034% and pH of 7, 0.185% and pH of 8, and 0.019% and pH of 5 had an important antibacterial effect by reducing the microbial count of *E. coli* from 12 CFU/μL to 5, 7, and 3 CFU/μL, respectively, while the sample with 0.185% concentration and pH of 8.5 did not inhibit the bacterial growth.

Selenium (Se) is another metal that can be incorporated into or coated on the PVC surface. It behaves as an efficient antimicrobial agent, but if used as nanoparticles, it promotes intracellular ROS in amounts directly related to the particle diameter [144]. As previously mentioned, ZnO NPs are considered less toxic than other metals or oxides and are a viable alternative to AgNPs, although they are also associated with ROS induction [145]. Photodynamic therapy and activation of TiO_2_ conduct to the electron holes production, a fact that is detrimental to bacterial ROS [97].

As an overall conclusion related to metal oxide NPs’ toxicity, a direct link is established between it and the crystallinity, size, surface area, and shape of the NPs [146]. Regarding the other antimicrobial approaches’ side effects, further research must be undertaken, and high levels of investment are needed to move the studies from in vitro and animal tests to clinical trials and to establish exactly the toxicological concerns.

The medical industry is still using silver-based coatings applied on ETTs because this metal is the most well-documented antibacterial material. To implement and design new antimicrobial coatings for medical device surfaces [147], some safety-design criteria must be met, which involve the analysis of biocompatibility, antimicrobial performance, and potential risk of producing antimicrobial resistance (AMR),. Silver coatings were involved in many pre-clinical and clinical trials from all the antibacterial approaches because they were introduced on the market in the USA. Although use of silver-based coating is limited by silver’s high costs [148], long-term stability, and toxicity that can be linked to systemic side effects [3,138,139], it is still the method of choice for many clinicians. Important concerns regarding the controlled release kinetics of antibiotics or antiseptics and the AMR-induction risks characterize other techniques such as ETT impregnation with biocides. It was noted that where antiseptics are used, toxicity and environmental issues are reported [149].

On the other hand, in the case of antimicrobial coatings based on natural compounds, some limitations can be underlined: their high costs, easy degradation by proteases, and in the case of phages, one can foresee as drawbacks their sensitivity to moisture and loss of properties if certain external conditions are met [150]. If we consider the passive approaches, manipulation of surface chemistry is difficult because it is based on complex protocols and expensive technologies [151]. Due to the important drawbacks mentioned above and the necessity of much more research in the field, silver-based coatings are the only ones that have received medical approval to be used in human treatment.

The disadvantages of the antimicrobial approaches described in this paper are summarized in Table 4.

## 3. Conclusions and Future Directions

Nowadays, pneumonia represents the second most prevalent nosocomial infection encountered in hospitals, and86% of all total cases are related to mechanical ventilation. In the United States of America, between 250,000 and 300,000 cases occur each year, representing an incidence rate of 5 to 10 cases per 1000 hospitalized patients [152,153]. Thus, manufacturing innovative antifouling and antimicrobial materials must be considered an important task. It is necessary to perform a combination of in vitro and in vivo studies to take a step in the direction of clinical trials, which are much more complex and expensive [154,155].

It is of utmost importance to develop materials with antimicrobial and antifouling characteristics using active or passive coatings or combinatorial methods. Much scientific research has reported in vitro studies based on different pathogen strains such as *S. aureus*, *P. aeruginosa*, *A. baumannii*, and *E. coli* and proved the efficiency of different proposed solutions for ETTs (Table 5). Unfortunately, many of the studies analyzed the biocidal characteristics of a polymeric material against only one pathogen strain. Tests showed that given species of *P. aeruginosa* exhibited an increased capability to develop biofilms. A limitation of the existing studies could be the need to analyze the effect of materials against a multispecies biofilm. This approach, combined with standardized cytotoxicity and antimicrobial evaluation methods and test conditions, can be linked to a much more realistic scenario that could make a transition to clinical trials. Van Charante et al. [156] investigated the microbial diversity and antimicrobial susceptibility in ETT biofilms related to mechanically ventilated COVID-19 patients. They stated that the most commonly identified pathogens were *P. aeruginosa*, *S. epidermis*, *E. faecalis*, *K. aerogenes,* and *C*. *albicans*, a fact that justified the choice of pathogen strains present in the in vitro studies described in our paper. Also, rarer proteobacteria such as *Paracoccus yeei, Neisseria baciliformis, Neisseria* spp*., Eikelnella* spp., and *Aureimonas* spp. were reported. The authors noticed that additionally to well-known potential respiratory pathogens (*P. aeruginosa* and *S. aureus*), opportunistic respiratory strains such as *Citrobacter koseri*, *Morganella morganii*, and *E. cloacae* were detected based on mass spectrometry analysis. The main conclusion of the study was that species that are normally part of the lung microbiome were combined with conventional and rare respiratory pathogens and led to a potential further complication of the preexistent infection. As a direct consequence, more complex in vitro studies are necessary to determine which types of antimicrobial coatings are suitable for different multispecies biofilms.

Table 5 summarizes the main species of pathogens investigated in the literature in relation to the antimicrobial methods presented in the paper.

Another limitation of the literature is associated with a need for in vivo analysis. These types of tests are necessary because they put in evidence very precisely the influence of the physiological media and location on the indwelling device. Of all the medical implants, ETTs are considered challenging because they are in contact with a large surface inside the human body. These medical implants are inserted along the esophagus, bronchi, and lungs, are placed in direct contact with different body fluids and tissue secretions, and are influenced by the gas flow and different pressures from the ETT cuff adjustment. Their insertion length can be up to 23 cm as a function of patient age, gender, or airway length [158]. Other risks that must be mentioned include the fact that bacterial contamination can occur at the moment of patient intubation, and prolonged intubation time can lead to biofilm formation and persistence.

The ETT surface modifications or coatings presented in this review paper put in evidence valuable data that permit scientists to reduce pathogens’ adherence to medical devices and even to kill bacteria and prevent biofilm formation and patient contamination. In active approaches based on metal coatings, antiseptic release, photo-based therapy, ceragenin, and bacteriophages, almost all the limitations associated with the passive technologies are overcome. Unfortunately, only silver-coated ETTs are on the market due to their high involvement in clinical trials, but concerns such as stability and cytotoxicity are a real problem. Other described solutions are still in the preclinical stage. Passive approaches include alterations of the patterning and chemical composition of the ETT surface, resulting in a nano-rough and hydrophobic surface that inhibits bacterial adherence and the aggregation of mucus secretions. Combinatorial technologies are another vital way to fight against VAP and represent a combination of active and passive approaches.

Usually, in the case of pediatric patients, bio-inspired antimicrobial coatings are considered one of the most suitable solutions. Aronson et al. [107] developed a proof-of-concept study in which they made an innovative coating that can release antimicrobial peptides that have an antibacterial effect on specific pathogens. This novel device can modulate the upper-airway microbiome and can prevent diseases such as subglottic stenosis. The endotracheal microbiome of intubated patients is unbalanced, and treating this problem with antibiotics can lead to antimicrobial resistance, which is not indicated in the children’s cases. A polymer coating that releases Lasioglossin-III was investigated in the study mentioned above. The efficiency of this peptide was tested against microbes, bacteria, and human microbiome (Table 5). It was concluded that this drug-eluting ETT could prevent biofilm formation and laryngotracheal stenosis and that it can be applied successfully in children [159]. Another method that can be used for children consists of chemical etching on the ETT surface due to a fungal lipase action, as described by Machado et al. [160]. This process generates a nano-roughened surface that hinders bacterial adhesion, as described in Section 2.2.1. Regarding other coating methods on ETTs that can be considered suitable and safe for children, published studies remain lacking.

This review paper aims to present and evaluate the existent solutions that can be applied to reduce and even prevent biofilm formation and increase scientists’ awareness of the global economic and social burdens of VAP. The chosen topic’s importance can also be seen from the fact that the global PVC ETT market is estimated to achieve about USD 2925.14 million by 2030, with a compound annual growth rate of 5.9% over the forecast period [161]. The market growth is directly proportional to increased surgery, disease burden, and technological discoveries. Uncoated ETTs are predominant on the market, with a revenue share of 32.10% in 2022. Coated ETTs are used in intensive care units to prevent VAP infections. Although a small number of producers that manufacture coated ETT are present on the market, the number of coated medical devices increased during the COVID-19 pandemic. The market size in 2021 was estimated at USD 159.4 million, with a market forecast value of more than USD 262 million and a growth rate of 5.1% over 2022–2031. It can be noticed that although conventional ETTs are cheaper than the coated ones, a similar dynamic of the market regarding growth rate until 2031 is foreseen. It is expected that coated ETTs will be beneficial in VAP prevention, and many hospitals worldwide will choose to use them to the detriment of classical ones [162].

From our point of view, coated or surface-modified ETTs are more efficient in ensuring adequate VAP prevention, although their cost is much higher than that of conventional ones. In this direction, we suggest the following next steps for research in the domain: complex in vitro studies, necessary to determine the antimicrobial and antibacterial efficiency of the coating or modified surface against a much larger number of pathogens encountered in biofilm composition, including rare respiratory bacteria; additional in vivo studies to prove the beneficial effect of the coating materials in cases of medium and long-term ventilation; less invasive coating or surface modifications based on bio-solutions for the development of ETTs dedicated to children and neonates; supplementary experimental tests to establish the biological range for each metal or oxide used in the ETT coating procedure; introduction in clinical trials of different antimicrobial coatings based on natural compounds and passive approaches regarding the surface chemistry modifications. These studies are necessary to gain a clear overview, and they must be accelerated by considering the gravity of the situation in hospitals worldwide. This fact is fundamental because each year, many more patients need to be intubated, and adequate solutions to increase patient comfort and care and to hinder the complications associated with pneumonia are a first-line problem.

## Figures and Tables

**Table 1:** Antimicrobial metal coatings that do not contain silver used on ETTs in in vitro or in vivo studies.

Antimicrobial Metal Coating	Coating Characteristics	Base Material/Antimicrobial feature	In Vitro/In Vivo Study	Remarks	Ref.
Zinc oxide (ZnO)	ZnO exhibits antimicrobial properties. Usually, ZnO nanoparticles are used due to low manufacturing costs, high surface-to-volume ratio, and enhanced stability. ZnO has high efficiency against Gram-positive and Gram-negative bacteria.	PVC/ZnO-nanoparticles (NPs)	In vitro (*S. aureus*)	Through the incorporation of ZnO NPs into PVC material, a reduction of biofilm formation by 55% was noticed after 72 h.	Seil et al. [91]
		PVC/ZnO NPs	In vitro (*S. aureus*)	A reduction of 87% in biofilm formation was reported after 24 h. An increase in the NPs’ concentration was linked to a high surface energy and roughness exhibiting a beneficial effect on bacteria reduction. The lower ZnO NP diameter led to increased antibacterial activity.	Geilich and Webster [92]
		Commercially available coated and uncoated ZnO NPs	In vivo (male BALB/c mice)	The animal models were exposed to endotracheal instillation with one dose (5 μg/mouse), and pulmonary inflammation was noticed. After a month of weekly exposure, a higher immune response was obtained in the case of uncoated nanoparticles.	Zhang et al. [93]
Selenium (Se)	Se NPs exhibit antioxidant, anti-oncological, and antibacterial properties [14]	PVC medical grade/Se NPs	In vitro (*S. aureus*)	A reduction of 80% was observed in the bacterial colonization process. The biofilm formation was reduced in comparison with a silver-coated ETT.	Tran and Webster [94]
Titanium dioxide (TiO_2_) combined with photodynamic therapy	Photodynamic therapy is based on a photosensitizer material, which can be activated at a given wavelength of light. It generates an active component that has antibacterial properties. TiO_2_ is chemically inert, stable, and exhibits photocatalytic properties by generating reactive oxidative species (ROS) when it is exposed to a wavelength of 385 nm [97]. TiO_2_ is efficient against both Gram-positive and Gram-negative bacteria.	Commercial ETT from PVC/TiO_2_ NPs (N-doped and commercially available standard anatase)	In vitro (*S. aureus* and *P. aeruginosa*)	In the case of light absence, no antimicrobial effects were observed. For fluorescent light irradiation, both types of coating exhibited almost the same effects on *P. aeruginosa*, and N-doped nanoparticles proved to be more efficient against *S. aureus*	Caratto et al. [95]
		PVC medical grade/iodine modified TiO_2_ NPs	In vitro (*E. coli*) and in vivo (pig model)	The modified TiO_2_ NPs presented photocatalytic antibacterial effects under visible light application. A decrease in bacterial attachment and biofilm formation was reported after 72 h. Reduced inflammation of the lungs was noticed in MV pigs after 72 h.	Deng et al. [96]

**Table 2:** Bio-inspired antimicrobial coatings that do not contain silver used on PVC ETTs.

Antimicrobial Compound	Pathogen	Test Type	Remarks	Ref.
Lasioglossin-III	*S. epidermis*, *S. pneumoniae*	In vitro	The design of a peptide-eluting tube based on a PLGA matrix with a continuous release of Lasioglossin-III proved to be efficient against planktonic bacterial development. There were no reported side effects on epithelial and fibroblast cell lines.	Aronson et al. [107]
Ceragenin CSA-131	*C. auris*, *K. pneumoniae*, *C. albicans*, *P. aeruginosa*, MRSA	In vitro, in vivo	The biofilm did not appear in the first 16 days. There was no reported multispecies biofilm formation for up to 3 days. In the pig animal model, no damages were observed to the trachea and lungs.	Hashemi et al. [40]
Sphingosine	*S. aureus*, *P. aeruginosa*, *A. baumanii*	In vitro, in vivo	In vitro efficacy against biofilm formation. In vivo tests evidenced the absence of inflammation and prevention of bacteria film.	Seitz et al. [113]
Phages	MDR *P. aeruginosa*	In vitro	The phage-coated samples were characterized by reduced bacterial colonization by a maximum of 3.2 log compared with uncoated samples.	Amankwah et al. [115]
Lecithin and cholesterol	*S. aureus*, *P. aeruginosa*	In vitro	After 8h, a decrease of 90% in biofilm formation was reported.	Jones et al. [116]

**Table 3:** Combinatorial active/antibacterial and passive/anti-biofouling materials for commercially available ETTs.

Main idea of the Combinatorial Strategy	Antimicrobial Compound	Pathogen	TEST TYPE	Remarks	Ref.
Metabolites such as fructose can enhance the antibiotics’ efficiency.	Surface with nanometric characteristics obtained after a combination of a fungal lipase and fructose.	*S. aureus*	In vitro	The nanoscale surface features obtained under the action of a fungal lipase determined a 45% decrease in *S. aureus* attachment after 24 h exposure. Soaking the unmodified surface ETT in fructose generated a 38% reduction. When the two strategies were combined, a decrease of about 60% in *S. aureus* attachment was reported.	Dumus et al. [121]
Natural polymer chitosan (CS), already used in the wound dressing domain, has a good interaction with metallic ions and nanoparticles.	CS-Ag nanoparticles @ polyacrylamide—gelatin composite	*S. aureus,* *P. aeruginosa*	In vitro (broncho-lung system); in vivo (pig model)	The complex nanocomposite coating exhibited important antibacterial properties during in vitro and in vivo tests. A reduction of 97% in lumen occlusion due to artificial mucus was observed. Antibiofouling characteristics were noticed due to reduced lumen occlusion. High biocompatibility of the coating evidenced by in vitro tests with fibroblasts	Wang et al. [132]
Zeolites are substances that are characterized by cavities and channels. They can trap metallic ions with antibacterial properties.	Zeolites with copper ions (CuZ) and D-Tyrosine (D-Tyr) solution.	MDR *A. baumannii*	In vitro	A reduction of 14% in immobilized cells was noticed after 24 h. The impregnation of composite system CuZ with D-Tyr (CuZ-Tyr) based on a synergistic effect proved to have an important antibacterial effect.	Milenković et al. [133]
	Zeolites with micronized silver (Ag-NZ) and D-Tyr.	MDR *A. baumannii*	In vitro	The Ag-NZ composites (1–15 wt.% Ag-NZ) were characterized by a decrease of up to 70% (4.4 log CFU) of immobilized pathogen compared with commercially available PVC. The samples Ag-NZ coated with D-Tyr (Ag-NZ-Tyr) exhibited a 100% bactericidal effect consisting of a 6.9 log CFU reduction against immobilized bacterial cells.	Milenković et al. [134]
Curcumin has an important photodynamic effect.	Curcumin combined with photodynamic action.	*S. aureus*, *P. aeruginosa*, *E. coli*	In vitro	Reductions of 95% (*S. aureus*), 72% (*E. coli*), and 73% (*P. aeruginosa*) in biofilm formation were noticed under the combined action of light and curcumin-functionalized ETT. The coating was still active after 6 light applications at a time interval of 24 h for 6 days. A pathogen decrease of 24% was reported.	Zangirolami et al. [135]
Hydrogel entrapped with nebulized drugs has antimicrobial and antibacterial effects.	Hydrogels made of hydroxyethylmethacrylate (HEMA): methacrylic acid (MAA) combined with nebulized gentamicin.	*S. aureus*, *P. aeruginosa*	In vitro	The most efficient combination was gentamicin-containing HEMA: MAA hydrogel. Another good combination was the 70:30 HEMA: MAA copolymer that presented a persistent effect against the tested pathogens at more than 20 days.	Jones et al. [136]
Antibacterial combined effect between chlorhexidine (CHX) and silver carbonate.	CHX and silver-based compound.	*A. baumannii,* *methicilin-resistant S. aureus MRSA*, *S.aureus*, *P. aeruginosa*, *Enterobacter aerogenes*	In vitro	The antiseptic-impregnated ETTs exhibited a reduced possibility of bacterial pathogen colonization compared with commercial ETTs. A reduction of 4–6 log of pathogen colonization of ETT after 5 days was reported.	Pacheco-Fowler et al. [101]
Combination of titanium dioxide (TiO_2_) and silver.	Silver, TiO_2_, and innovative metallic alloy Degussa	*S. aureus*, *P. aeruginosa*	In vitro	No positive effect was reported against *S. aureus*. Regarding *P. aeruginosa*, the silver combined with TiO_2_ reduced film growth after 24 h, while the combination of Degussa and TiO_2_ presented a diminution of pathogen growth after 48 h.	Tarquinio et al. [137]

**Table 4:** Main disadvantages of the antimicrobial approaches used to improve the antibacterial efficiency of ETTs.

Antimicrobial Approach	Strategy	Disadvantage	Selective Ref.
Active antimicrobial coatings	Antimicrobial metal coatings	Toxicity of metal ions or nanoparticles; propensity to induce bacterial resistance.	Setyawati et al. [138]; Delawal et al. [139]
	Antimicrobial coatings based on biocide impregnation	Difficult control of release kinetics of active substances; development of antimicrobial resistance and induced drug-resistant strains when antibiotics are used; toxicity and environmental problems linked to biocides in a dose-dependent manner.	Ahonen et al. [149]; Alves et al. [14]
	Bio-inspired antimicrobial coatings	Proteolytic degradation; cytotoxicity and hemolysis concerns regarding AMPs; high costs of AMPs; the use of phages is characterized by moisture sensitivity and deactivation under certain conditions.	Alves et al. [14]; Hosseinidoust et al. [150]
Passive coatings	Nanomodified surfaces	Surface modifications are obtained through surface chemistry manipulation, which is a difficult process, requiring the development of complex protocols and specific technological methodologies.	Alves et al. [14]; Machado et al. [122]
	Hydrophilic/hydrophobic surface modification	High costs when plasma treatment is involved.	Jacobs et al. [151]
	Micropatterned surface modifications	Structural durability and stability; biocompatibility, environmental concerns, high costs.	Mann et al. [63]; May et al. [128]
Combinatorial materials	Active and passive strategies	Combine the disadvantages of different involved strategies previously underlined.	Alves et al. [14]; Barnes et al. [3]

**Table 5:** Main pathogen strains related to antimicrobial coatings applied on ETT surfaces.

Antimicrobial Strategy	Antimicrobial Feature	Pathogen Strain	Selective Ref.
Active/Antimicrobial metal coatings	Ag	*A. baumanii, C. albicans, K. pneumoniae, P. aeruginosa, Enterococcus faecalis,* MRSA (methicilin-resistant *S. aureus*)*, S. aureus, E. coli*	Lethongkam et al. [85]; Loo et al. [87]; Jiang et al. [88]
	NMA (Au-Ag-Pd)	*Enterococci spp., Neisseria spp., Haemophiles parainfluenza, Streptococcus, Staphylococci*	Björling et al. [64]; Tincu et al. [90]
	ZnO, TiO_2_, Se	*S. aureus, P. aeruginosa, E. coli*	Seil and Webster [91]; Caratto et al. [95]; Deng et al. [96]; Tran and Webster [94]
Active/Biocidal impregnation	Gardine and gendine, hexetidine	*A. baumannii*, *E. cloacae*, *C. albicans*, *K. pneumoniae*, MRSA, *P. aeruginosa, S. aureus*	Raad et al. [100]; Jones et al. [102]
	Poly(lauryl acrylate)-based nanocapsules with eugenol or clove oil, styrylbenzene-based (BCP3)	MRSA, *K. pneumoniae,* MSSA (methicilin-sensitive *S. aureus*)*, P. aeruginosa*	Venkateswaran et al. [103]; Ozcelik et al. [157]
Active/Bio-inspired antimicrobials	Lasioglossin-III, ceragenin CSA-131, cholesterol and lecithin, sphingosine, phages	*S. epidermis*, *S. pneumoniae*, *C. albicans*, *C. auris*, *K. pneumoniae*, *P. aeruginosa*, MRSA, *S. aureus*,* A. baumannii*, MDR (Multidrug resistant) *P. aeruginosa*	Aronson et al. [107]; Hashemi et al. [40]; Jones et al. [116]; Seitz et al. [113]; Amankwah et al. [115]
Passive/Nanomodified surfaces	Fungal lipase	*S. aureus, P. aeruginosa*	Machado et al. [122]; Machado and Webster [123]
Passive/Hydrophilic/hydrophobic surface modification	Plasma treatments	*P. aeruginosa*	Triandafillu et al. [125]
Passive/micropatterned surfaces	Sharklet pattern design	*A. baumannii*, *K. pneumoniae*, MRSA, *E. coli*, *P. aeruginosa*	May et al. [128]; Mann et al. [63]
Combinatorial materials	Chitosan—AgNPs—polyacrylamide, fungal lipase and fructose, chlorhexidine and silver carbonate, copper and zeolite, hydrogels with nebulized gentamicin, curcumin and photodynamic effect	*P. aeruginosa, S. aureus, A. baumannii, Enterobacter aerogenes,* MRSA, MDR. *A. baumannii, E. coli*	Wang et al. [132]; Durmus et al. [121]; Pacheco-Fawler et al. [101]; Milenković et al. [133]; Jones et al. [136]; Zangirolami et al. [135]

## Data Availability

Not applicable.
